# The influence on survival of delay in the treatment initiation of screening detected non-symptomatic breast cancer

**DOI:** 10.1038/s41598-019-46736-1

**Published:** 2019-07-15

**Authors:** Yan Li, Yidong Zhou, Feng Mao, Jinghong Guan, Yan Lin, Xuejing Wang, Yanna Zhang, Xiaohui Zhang, Songjie Shen, Qiang Sun

**Affiliations:** Department of Breast Surgery, Peking Union Medical College Hospital, Peking Union Medical College, Chinese Academy of Medical Sciences, Beijing, 100730 P.R. China

**Keywords:** Breast cancer, Breast cancer

## Abstract

We aimed to determine whether the detection-to-treatment interval of non-symptomatic breast cancer is associated with factors that can predict survival outcomes. A retrospective review of the Breast Surgery Department Database at Peking Union Medical College Hospital (PUMCH) was performed, and a total of 1084 non-symptomatic invasive breast cancer patients were included. The findings revealed that detection-to-treatment interval was significantly longer for women who were older (*p* = 0.001), lived in rural areas (*p* = 0.024), had lower education (*p* = 0.024), and had detection in other institutions (*p* = 0.006). Other sociodemographic and clinicopathological characteristics were not associated to longer interval. A median follow-up of 35 months (range: 6–60 months) was carried out and a long delay at more than 90 days did not significantly decrease the DFS (univariate, *P* = 0.232; multivariate, *P* = 0.088). For triple negative breast cancer, there was a worse DFS if the interval was longer than 90 days both in multivariate analysis (hazard ratio [HR] = 3.40; 95% CI, 1.12–10.35; *P* = 0.031) and univariate analysis (HR = 2.86; 95% CI, 1.03–7.91; *P* = 0.042). Further studies on care before initial treatment of non-symptomatic breast cancers are warranted.

## Introduction

Long intervals between detection of breast cancer and therapy initiation may affect the prognosis. A delay may lead to disease progression or treatment complications. There are many factors that may contribute to the delay of treatment and, although many studies have assessed how this may have influenced recurrence and survival, the results have been conflicting^1–11^. A British study showed that treatment delays, when defined as having treatment symptoms for more than 12 weeks, adversely affected the prognosis^10^. Another study in Asian breast cancer patients indicated that delays of more than 6 months resulted in worse disease-free survival^4^. However, other studies have found no significant association between time-to-treatment after detection and the pathological diagnosis of breast cancer and survival rates^3,6,8,9,11^.

Breast cancer is the most frequent type of cancer in Chinese women and the age- standardized rate is 21.6 cases per 100 000 women^12^. As imaging screening is becoming more and more common, a large number of early breast cancers have been detected by screening rather than obvious symptoms. However, there has been scarcity of information on delay in the treatment initiation of screening detected non-symptomatic breast cancer. In this study, we investigated the demographic and clinicopathological factors associated with delay of the initiation of treatment after screening and the detection of non-symptomatic breast cancer, and we assessed the impact of delay of treatment on patient survival.

## Results

### Patient characteristics

A total of 1084 non-symptomatic breast cancer patients who were screened and subsequently shown to have breast cancer and who underwent definitive surgery for invasive breast cancer at Peking Union Medical College Hospital (PUMCH) were included in this study. All participants were female and of Asian descent. Among them, 1056 cases had surgery as the initial treatment, and 28 cases had chemotherapy or endocrine therapy as the initial treatment. In 1049 cases, diagnostic open excisional biopsy and intraoperative frozen section examination were performed, followed by definitive surgeries immediately. Their median interval between screening detection date and the date of initial treatment was 38 days (range: 3–282 days). A total of 792 (73.06%) women received the first treatment within 90 days of detection, and 292 (26.94%) received their first treatment more than 90 days after detection.

### Factors predicting the interval before treatment initiation

Various factors were associated with a longer interval between screening and detection and the initiation of treatment (Table 1). The interval was significantly longer for women who had an older age (47.84 ± 13.67 vs. 44.92 ± 11.71, *p* = 0.001), those who lived in the rural areas (*p* = 0.024), those who had lower education (*p* = 0.024), and those who had the screening detection in other institutions (*p* = 0.006). Menstrual status and marital status were not associated with longer intervals between detection and the initiation of treatment (*p* > 0.05). There was no significant association between breast cancer family history and comorbid conditions and longer intervals to treatment (*p* > 0.05).

**Table 1:** Patient characteristics within subgroups.

Characteristic	Delayed days ≤90 (n = 792)	Delayed days >90 (n = 292)	P*
Age, years			0.001
Mean ± SD	47.84 ± 13.67	44.92 ± 11.71	
Menstrual status, No. (%)			0.346
Premenopausal	443 (55.93)	175 (59.93)	
Postmenopausal	309 (39.02)	100 (34.25)	
Unknown	40 (5.05)	17 (5.82)	
Residence, No. (%)			0.024
Urban	446 (56.31)	142 (48.63)	
Rural	346 (43.69)	150 (51.37)	
Education, No. (%)			0.021
≥High school	498 (62.88)	161 (55.14)	
<High school	294 (37.12)	131 (44.86)	
Marital status, No. (%)			0.875
Married	583(73.61)	215(73.63)	
Unmarried	115(14.52)	45 (15.41)	
Other	94(11.87)	32 (10.96)	
Screening detection places, No. (%)			0.006
PUMCH	389 (49.12)	116 (39.73)	
Other institutions	403 (50.88)	176 (60.27)	
Tumor size, No. (%)			0.482
T1	680 (85.86)	252 (86.30)	
T2	96 (12.12)	31 (10.62)	
T3	16 (2.02)	9 (3.08)	
Tumor grade, No. (%)			0.071
G1	261 (32.95)	75 (25.68)	
G2	314 (39.65)	129 (44.18)	
G3	217 (27.40)	88 (30.14)	
Molecular subtype, No. (%)			0.095
Luminal A	372 (46.97)	151 (51.71)	
Luminal B	182 (22.98)	70 (23.97)	
Triple Negative	158 (19.95)	55 (18.84)	
Her-2+	80(10.10)	16(5.48)	
Axillary node metastasis, No. (%)			0.360
No	682 (86.11)	245 (83.90)	
Yes	110 (13.89)	47 (16.10)	
Comorbid conditions, No. (%)			0.198
Yes	146(18.43)	64(21.92)	
No	646(81.57)	228(78.08)	
Breast cancer family history, No. (%)			0.789
Yes	58(7.32)	20(6.85)	
No	734(92.68)	272(93.15)	

SD, standard deviation; PUMCH: Peking Union Medical College Hospital. *Categorical data were compared using a two-tailed chi-squared test. Quantitative data were compared by Student’s t-test. Differences were considered significant at *P* < 0.05.

Pathologically, tumor size, tumor grade, molecular subtype, and axillary lymph node involvement were not associated with longer intervals between detection and comorbid conditions and the initiation of treatment (*p* > 0.05).

### Survival analysis between groups with different intervals (≤90 days vs. >90 days)

**Figure 1:**
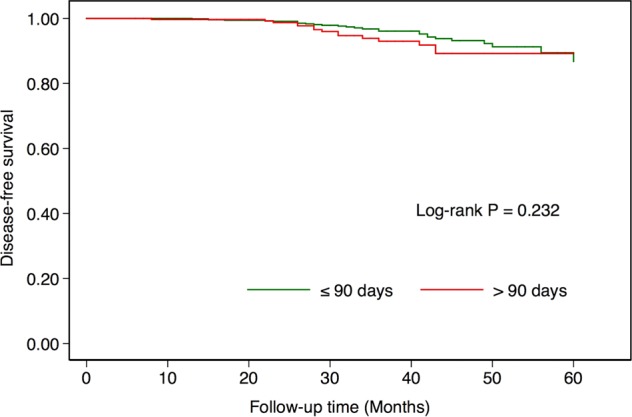
Kaplan-Meier survival curves of disease-free survival (DFS) by interval between screening detection and treatment initiation (≤90 days vs. >90 days).

The median follow-up time was 35 months (range: 6–60). In the group with an interval within 90 days, the 3-year Kaplan-Meier estimates for DFS was 96.1% (Fig. 1). In the group with an interval longer than 90 days, the 3-year Kaplan-Meier estimate for DFS was 93.0% (Fig. 1). The log-rank comparison indicated no significant difference in DFS (*P* = 0.232) in the group with an interval less than 90 days compared to the group with an interval longer than 90 days.

Independent prognostic factors for DFS included in the multivariate analysis were age, tumor size, nodes status, tumor grade, molecular subtype, and interval from screening detection to the initiation of treatment (Table 2). Long delays before treatment of more than 90 days did not significantly decrease the DFS (Hazard Ratio [HR] = 1.74; 95% CI, 0.92–3.30; *P* = 0.088) after adjusting for other prognostic factors.

**Table 2:** Cox proportional hazards regression model analysis of disease-free survival.

Factor	Disease-free survival			
	HR	95% CI	P*	
Age	(Continuous)	1.00	0.99–1.03	0.426
Tumor size	T1	Reference		
	T2	1.33	0.57–3.10	0.507
	T3	2.35	0.76–7.28	0.137
Grade	G1	Reference		
	G2	0.72	0.34–1.54	0.399
	G3	1.06	0.51–2.21	0.879
LN	No metastasis	Reference		
	Metastasis	2.40	1.21–4.77	0.012
Molecular subtype	Luminal-A	Reference		
	Luminal-B	1.77	0.66–4.80	0.258
	TNBC	4.55	1.98–10.45	0.000
	Her-2+	8.47	3.44–20.86	0.000
Interval from detection to treatment	≤90 days	Reference		
	>90 days	1.74	0.92–3.30	0.088

HR: hazard ratio, CI: confidence interval, LN: lymph node, TNBC: triple negative breast cancer. *Differences were considered significant at *P* < 0.05.

### Stratification by molecular subtype

In addition, we investigated the effect of interval from screening detection to treatment initiation on survival by analyzing breast cancer molecular subtypes. In the univariate analysis, patients receiving their initial treatment more than 90 days from detection had a worse DFS than those who received treatment at an earlier time in TNBC (HR = 2.86; 95% CI, 1.03–7.91; *P* = 0.042), but not in luminal-A (HR = 1.34; 95% CI, 0.33–5.39, *P* = 0.672), luminal-B (HR = 0.87; 95% CI, 0.16–4.54; *P* = 0.874), or HER2 + tumors (HR = 2.14; 95% CI, 0.56–8.15; *P* = 0.262) (Table 3). After adjustment for age, the results were maintained for tumor grade, tumor size, and node status. For those patients with TNBC disease, delayed treatment had a significant influence on DFS (HR = 3.40; 95% CI, 1.12–10.35; *P* = 0.031). In contrast, patients with HER2 + , luminal-A, and luminal-B tumors, and those who received delayed treatment had a HR of 2.65 (95% CI, 0.57–12.31), 1.18 (95% CI, 0.26–5.28), and 0.76 (95% CI, 0.08–7.19) for DFS, respectively, when compared to those who started initial treatment earlier (Table 3).

**Table 3:** Influence of interval from detection to treatment initiation on disease-free survival in different molecular subtypes.

Molecular subtype	Hazard for DFS (≤90 days vs. >90 days)		
	Univariate *P* (log-rank)	Multivariate HR* (95% CI)	Multivariate *P*
Luminal-A (n = 523)	0.672	1.18 (0.26–5.28)	0.828
Luminal-B (n = 252)	0.874	0.76 (0.08–7.19)	0.809
TNBC (n = 213)	0.042	3.40 (1.12–10.35)	0.031
Her-2(+) (n = 96)	0.262	2.65 (0.57–12.31)	0.212

*Adjusted for age, tumor size, tumor grade and nodes status. HR, hazard ratio; CI, confidence interval; TNBC, triple negative breast cancer.

## Discussion

Breast cancer delay, which is defined in the literature as more than 90 days between symptom detection and the initiation of definitive breast cancer treatment (radiation therapy, surgery, chemotherapy, endocrine therapy, or targeted therapy), is a major medical problem in low- and middle-income countries^1,13–15^. Some studies have divided this problem into two parts: (1) patient delay defined as the time between symptom manifestation and the first medical consultation, and (2) provider delay defined as the interval between the first medical consultation and the initiation of definitive treatment^1,13,16^. However, this classification is over-simplified and arbitrary, because patient factors and health care provider factors are always intertwined in the whole process from the discovery of breast abnormality to treatment initiation. On the one hand, patients’ age, socioeconomic status, psychological and cognitive factors, and lack of medical knowledge can all exert an important influence on almost all phases of care-seeking process. On the other hand, the accessibility and efficiency of health care system are not always satisfactory in developing countries, which are barriers for breast cancer patients to obtain timely diagnosis and treatment^17–19^. Along with this total breast cancer delay, some studies also focus on other intervals: detection-to-diagnosis interval, diagnosis-to-treatment interval, and in-hospital interval (the time between the first consultation and the beginning of treatment)^20^.

A number of studies have determined whether a delay in diagnosis and treatment has an adverse impact on the survival rate of breast cancer^6,8,10,11,21,22^. A meta-analysis that included 38 studies found delays of 3–6 months were significantly associated with worse survival^23^. Two population-based cohort studies from South Korea indicated that longer intervals between diagnosis and treatment initiation were associated with poor overall survival in breast cancer^24^. However, Mujar *et al*. reported that delays over two months in time to treatment had no impact on breast cancer survival rates^25^. Yoo *et al*. found an interval between pathological diagnosis and treatment initiation of 60 days or less did not adversely affect DFS of breast cancer^11^. In addition to the conflicting conclusions, most of the previous studies assessed the effects of symptom-to-treatment interval or diagnosis-to-treatment interval, and there has been no study focusing on detection-to-treatment interval of non-symptomatic breast cancers. With the development of medical service and tumor screening, more and more non-symptomatic early-stage breast cancers have been detected by imaging approaches like mammography and ultrasound^26^. For these breast cancer patients, whose diseases were mostly of early stages, it was unclear whether the detection-to-treatment interval was associated to any sociodemographic or clinicopathological factors, or whether it could predict survival outcomes.

This study focused on the delay of treatment initiation of screening detected non-symptomatic breast cancer. In China, core needle biopsy is not routinely used to make pathologic diagnosis for breast disease because of economic and technical reasons. In most parts of China, diagnostic open excisional biopsy and intraoperative frozen section examination are commonly used to identify the pathology of primary tumors in more than 80% of breast cancer cases^27^. Similarly, in our routine procedure, we will first perform open excisional biopsy for suspicious breast lesions. If the frozen section reveals malignancy, a definitive surgery will be carried out immediately. In this scenario, pathologic diagnosis and definitive surgery are performed on the same day. For this study, 96.8%(1049/1084) of the total cases were like this. We have reason to believe that, to a great extent, “detection-to-treatment interval” can also reflect “detection-to-diagnosis interval” in this study.

This study suggested that detection-to-treatment interval longer than 90 days was significantly more common in women with older age. Previous studies showed that older women more often had a delay in treatment after a diagnosis of breast cancer, because they may attribute the early signs of tumor to the impairs caused by ageing and menopause^21,28^. In addition, older women are more likely to believe in fatalism, thinking that how long they can live is predestined and can hardly be changed^21^. Some studies also confirmed that, in older patients, a delay could be the result of negative attitudes toward seeking care and fear regarding the consequences of the diagnosis of breast cancer^29^.

We discovered that patients living in rural areas were more likely to postpone breast cancer treatment. There was a large urban-rural disparity worldwide in the diagnosis and treatment of breast cancer, and it is especially severe in China^30,31^. In rural areas, many patients lack health insurance coverage, and their incomes are not always enough for the high medical expense of cancer care. This low socioeconomic status is a barrier to timely health care access^1,13,17^. In addition, the quality and accessibility of health care system are not satisfactory in the countryside of China. Because of the lack of high-quality medical service, those who live in rural areas usually must spend more effort and time arranging visits for diagnosis and treatment because medical services in cities with high population numbers are usually much better than those of the countryside^12^.

In our study, higher education was associated with shorter delays. It seems that people who are highly educated employ more effective and on-time measures to take care of their health problems in comparison with less-educated people^28,32,33^. It is also important to keep in mind that complementary and alternative medicine (CAM), which includes herbal medicine, acupuncture, and massage, is very popular among less-educated people, especially in Asian countries. It was reported that use of CAM was associated with delays in the detection, diagnosis, and treatment of breast cancer^34^. However, the efficacy of CAM is uncertain for breast malignancy.

Another factor contributing to the interval was whether the tumor was screening detected in other institutions. In China, the appointment and referral system of medical service has not been well established^35^. As a result, on screening detection of breast cancer in local hospital where the medical service is not satisfactory, patients usually have to go to high-volume hospitals in big cities such as Beijing and Shanghai. However, the waiting time is usually very long due to the long queues in every step of health care seeking^17,26^. That is a very complicated, expensive, and time consuming process which will inevitably lead to delay of treatment.

This study established that a delay in the initiation of treatment at a cut-off point of 90 days after screening detection had no influence on DFS in non-symptomatic breast cancer, by both univariate and multivariate analyses. However, subgroup analysis showed that patients receiving initial treatment more than 90 days from screening detection had a worse DFS than those who received initial treatment at an earlier time in TNBC, but not in other molecular subtypes. There are no previous studies that have reported on the influence of treatment delay on patient survival considering various molecular subgroups. However, a retrospective review of TNBC patients suggested that there was a tendency toward worse overall survival with delays of more than 90 days. This result, however, was not statistically significant (*p* = 0.06)^6^. TNBCs have a higher proliferation rate and nuclear grade compared to other molecular subtypes. Both of these attributes contribute to biologic aggressiveness and increased risk of recurrence^36^. According to our results, the waiting time before initial treatment of TNBC patients should be shortened to obtain a better outcome.

There are a number of limitations in this study. First, and most importantly, caution should be used with retrospective studies because of the potential for confounding factors to influence the results. However, a prospective study that evaluated the effect of delay when treating breast cancer would be unrealistic and unethical. Second, our sample size may not have been large enough to draw a definitive conclusion regarding the impact of delays to the initiation of treatment on survival, especially for longer intervals. Actually, in this study we also tried 120 days or 150 days as the cut-off point, however, the number of cases in the longer delayed group would be so small that it was hard for a statistical analysis. The follow-up is also relatively short. Thirdly, because most of our patients received breast surgery followed by adjuvant chemotherapy, the neoadjuvant rate was low in our study. Furthermore, because we were focusing on the screening detected non-symptomatic breast cancers, the tumor stage was more likely to be early. In this cohort, more than 85% of the tumors were smaller than 2 cm, and less than 20% of them had node metastases. Future studies should also carefully explore the effect of delay to treatment in tumors with more advanced stages.

In conclusion, our findings revealed that the delay interval between detection and treatment initiation of non-symptomatic breast cancer was significantly associated to age, residential place, education, and place of screening detection. There is no difference in disease-free survival whether the interval is longer than 90 days or not. However, in TNBC patients, a worse survival seems to be possible when the interval exceeds 90 days. Further investigation is needed to assess additional predictors influencing delay and its effect on survival.

## Methods

### Ethics statement

Approval was obtained from the independent ethical committee/institutional review board of PUMCH. All included study patients provided written informed consent. We obtained permission of PUMCH to collect data from the Breast Surgery Department Database. Our methods were carried out in accordance with the relevant regulations and guidelines.

### Patients

We retrospectively reviewed the Breast Surgery Department Database at PUMCH. We included patients who underwent definitive surgery for invasive breast cancer at PUMCH between January 2013 and January 2016. All the included cases were non-symptomatic and were screening detected by imaging approaches (mostly by mammography, ultrasound, or both). All these patients started their initial treatment at PUMCH, and those who reported clinical symptoms and who underwent palliative operations for Stage IV diseases were dismissed from the study. All the eligible patients derived from the database had adequate medical and sociodemographic information and were followed up. The last follow-up date was January 19, 2018.

### Study design

We collected data on patients’ tumor characteristics and sociodemographic information, including age, residential places, menstrual status, marital status, educational status, comorbidities, place of screening detection, tumor grade, axillary lymph node involvement tumor size, molecular subtypes, and family history of breast cancer. The interval between screening detection and the initiation of treatment was defined as the time between the first detection date to the date of initial treatment, which might be chemotherapy, surgery, or endocrine therapy. An interval cutoff point of 90 days was used to assess the association with survival.

The endpoint of this study was disease-free survival (DFS). DFS was calculated from the time of the initiation of treatment to recurrence, second primary cancer, or death without evidence of recurrence or a second primary cancer. Breast cancer recurrence included distant metastases and locoregional recurrence.

### Statistical analysis

Categorical data were compared with a two-tailed chi-squared test. Quantitative data were analyzed with a Student’s t-test. The Kaplan-Meier method was used to determine the disease-free survival (DFS). We used two-sided log-rank tests for time-to-event endpoints. Multivariate survival analysis was performed using a multivariate Cox proportional hazards regression model adjusting for clinicopathological factors that are known to affect patient survival, including age, lymph node metastasis, tumor size, histologic grade, and molecular subtype. Significance was set at *P* < 0.05. Statistical analyses were performed using the statistical software package STATA (version 14.0, Texas, USA).

## Data Availability

All data that was obtained and analyzed during this study are included in this article.
